# Hesperidin Nanoformulation: A Potential Strategy for Reducing Doxorubicin-Induced Renal Damage via the Sirt-1/HIF1-α/VEGF/NF-κB Signaling Cascade

**DOI:** 10.3390/ph17091144

**Published:** 2024-08-30

**Authors:** Fatemah A. Alherz, Thanaa A. El-Masry, Ghaleb A. Oriquat, Engy Elekhnawy, Nora Hamad Al-Shaalan, Mohamed M. S. Gaballa, Enas I. El Zahaby, Maysa M. F. El-Nagar

**Affiliations:** 1 Department of Pharmaceutical Sciences, College of Pharmacy, Princess Nourah Bint Abdulrahman University, P.O. Box 84428, Riyadh 11671, Saudi Arabia; faalherz@pnu.edu.sa; 2 Department of Pharmacology and Toxicology, Faculty of Pharmacy, Tanta University, Tanta 31527, Egypt; thanaa.elmasri@pharm.tanta.edu.eg; 3 Faculty of Allied Medical Sciences, Al-Ahliyya Amman University, Amman 19328, Jordan; goreqat@ammanu.edu.jo; 4 Pharmaceutical Microbiology Department, Faculty of Pharmacy, Tanta University, Tanta 31527, Egypt; engy.ali@pharm.tanta.edu.eg; 5 Department of Chemistry, College of Science, Princess Nourah Bint Abdulrahman University, P.O. Box 84428, Riyadh 11671, Saudi Arabia; nhalshaalan@pnu.edu.sa; 6 Department of Pathology, Faculty of Veterinary Medicine, Benha University, Toukh 13736, Egypt; mohamed.gaballah@fvtm.bu.edu.eg; 7 Department of Pharmaceutics, Faculty of Pharmacy, Delta University for Science and Technology, Gamasa 35712, Egypt; enas.elzahabi@deltauniv.edu.eg

**Keywords:** doxorubicin, hesperidin nanoparticles, nephrotoxicity, oxidative stress, inflammation, Sirt-1

## Abstract

Hesperidin (Hes) functions as a strong antioxidant and anti-inflammatory to guard against damage to the heart, liver, and kidneys. Nevertheless, due to its restricted solubility and bioavailability, a delivery method is required for it to reach a specific organ. In this study, ion gelation was used to synthesize a chitosan/hesperidin nanoformulation. Numerous characterization techniques, such as zeta potential, particle size, XRD, TEM, SEM, and FTIR analyses, were used to corroborate the synthesis of hesperidin nanoparticles (Hes-NPs). Male albino mice were given a pretreatment dose of 100 mg/kg, PO, of Hes or Hes-NPs, which was administered daily for 14 days before the induction of doxorubicin nephrotoxicity on the 12th day. Kidney function (urea and creatinine levels) was measured. Lipid peroxidation (MDA) and antioxidant enzyme (CAT and SOD) activities were estimated. TNF-α, IL-1β, and VEGF content; histopathological examination of kidney tissue; and immunohistochemical staining of NF-κB, Caspase-3, BAX, Bcl-2, and TGF-β1 were evaluated. The gene expressions of *Sirt-1*, *Bcl-2*, *VEGF*, *HIF1-α*, and *Kim-1* were also considered. The results showed that pretreatment with Hes or Hes-NPs reduced doxorubicin’s nephrotoxic effects, with Hes-NPs showing the greatest reduction. Kidney enzyme and MDA content were lowered in response to the Hes or Hes-NP pretreatment, whereas antioxidant enzyme activities were increased. Hes or Hes-NP pretreatment suppressed the levels of TNF-α, IL-1β, VEGF, NF-κB, Caspase-3, BAX, and TGF-β1; however, pretreatment increased Bcl-2 protein levels. Furthermore, the gene expressions of *Sirt-1*, *Bcl-2*, *VEGF*, *HIF1-α*, and *Kim-1* were considerably higher with Hes-NP than with Hes treatment. These results suggest that Hes-NP treatment might reduce DOX-induced nephrotoxicity in mice via modulating Sirt-1/HIF1-α/VEGF/NF-κB signaling to provide antioxidant, anti-inflammatory, and anti-apoptotic effects.

## 1. Introduction

Kidney injury is a significant problem for health systems worldwide due to its high morbidity and mortality rates. Acute kidney injury involves a sudden worsening of the overall function of the kidney [1]. This usually occurs as a result of exposure to certain causative agents that have detrimental impacts on kidney structures. Such causative agents could be chemical, physical, or biological [2]. Among the most common chemicals that induce kidney injury is doxorubicin (DOX).

DOX is a wide-spectrum anthracycline frequently employed for managing various types of malignancies, such as leukemia, lymphomas, and solid tumors [3]. Nevertheless, the clinical usage of DOX is limited due to its toxic effects on many organs, such as the liver, kidney, heart, testis, lung, and nervous system. The kidney, in particular, is highly affected, owing to its high blood perfusion, as well as its role in drug metabolism. Therefore, alleviating DOX-triggered nephrotoxicity is a significant concern that has drawn the attention of various researchers [4].

Natural compounds have been widely investigated for their different pharmacological potentials [5], and, as a therapeutic approach, they have been implemented in combination with DOX to mitigate their toxic effects on the body’s organs [6].

Hesperidin (Hes) is a polyphenolic flavonoid that is part of a class of phytochemical compounds known for their various pharmacological actions, including anti-inflammatory, antioxidant, and anticancer effects [7]. Several clinical studies have documented the beneficial effects of Hes in managing hepatic, renal, and respiratory problems. However, it suffers from the great disadvantage of low water solubility and low oral bioavailability [8,9].

Hes is classified as a class II drug, and its rate-limiting step to reach systemic circulation (bioavailability) is water solubility. So, one technique to enhance the solubility of this class is its transformation to the nano-scale [10]. The transformation of Hes into the nano-sized amorphous state means an increased exposed surface area which would significantly enhance the dissolution rate according to the Noyes–Whitney equation dc/dt = K (Cs-C). The assessment of bioequivalence for a drug intended for target/local action and a poor systemic absorption drug can be conducted through pharmacodynamics bioequivalence testing (PD) instead of a pharmacokinetic test (PK) [11].

The estimation of Hes in plasma is considered a troublesome issue; a validated LC/MS/MS method is required with both a linearity range and suitable selectivity to detect the drug during its absorption, distribution, metabolism, and excretion ADME, especially during the absorption and elimination phases [12].

This study focused on studying PD as evidence for the improvement in the solubility of Hes and, consequently, Hes bioavailability. Also, we aimed to create a nanoformula of Hes to increase its oral bioavailability and therapeutic efficacy.

Chitosan (Cs) was selected based on several studies indicating its role in enhancing the solubility of different drugs, and especially due to being a natural biodegradable in addition to its mucoadhesion properties which would enable the sustained and prolonged effect of the formulation [13]. It is produced by deacetylating chitin [14,15]. Because of its high biocompatibility, chitosan has been used in a wide range of applications, including biomaterial development, tissue engineering, and the formulation of antibacterial, antifungal, anticancer, anti-inflammatory, and antioxidant agents [15,16,17,18].

Both the amino and hydroxyl groups of Cs are essential for their unique properties, such as permeation enhancement, controlled drug release, in situ gelation, and antimicrobial, anti-cancer, and wound-healing properties. Cs’s cationic nature contributes to its mucoadhesive properties; its amino groups create non-covalent bonds with mucin and adhere to the mucosal surface, a process known as mucoadhesion [19].

The main antioxidant constituent of Hes is its phenolic compounds, and previous studies illustrated the success of Cs in enhancing the bioavailability of tea polyphenols [20].

The release of encapsulated therapeutic molecules from Cs nanoparticles governs several release mechanisms such as swelling, diffusion, and erosion. Being a positively charged molecule, Cs significantly interacts with the mucous membrane, opens the tight junctions (TJs) between epithelial cells by reducing the electrical resistance, and promotes passage via the mucosal cells, thereby improving the permeation of encapsulated drugs in the nanoformulation. Cs inhibits the P-glycoprotein (P-gp) efflux transporter of epithelial cells, thus significantly facilitating the encapsulation of molecules by the paracellular transport mechanism [21]. For the manufacture of chitosan nanoparticles, multiple approaches have been examined, including polyelectrolyte complexation [22], covalent cross-linking [23], and ionotropic gelation [24].

Ionotropic gelation was selected because of its gentle and aqueous processing conditions, non-toxic chemicals, and ease of production, making it suited for clinical upscaling [25]. Likewise, chitosan nanoparticles have previously been shown to successfully deliver medications in vivo, such as insulin [26], cyclosporin A, and an immunosuppressant [27].

It has been documented that the principal pathophysiological mechanism of DOX-triggered kidney injury involves the induction of inflammation, apoptosis, and oxidative stress [28]. Thus, in the current investigation, we aimed to elucidate the potential role of hesperidin nanoparticles (Hes-NPs) in ameliorating the nephrotoxicity induced by DOX and to reveal its probable mechanism.

## 2. Results

### 2.1. Particle Size, Zeta Potential, and Entrapment Efficiency of Hes-NPs

**Figure 1:**
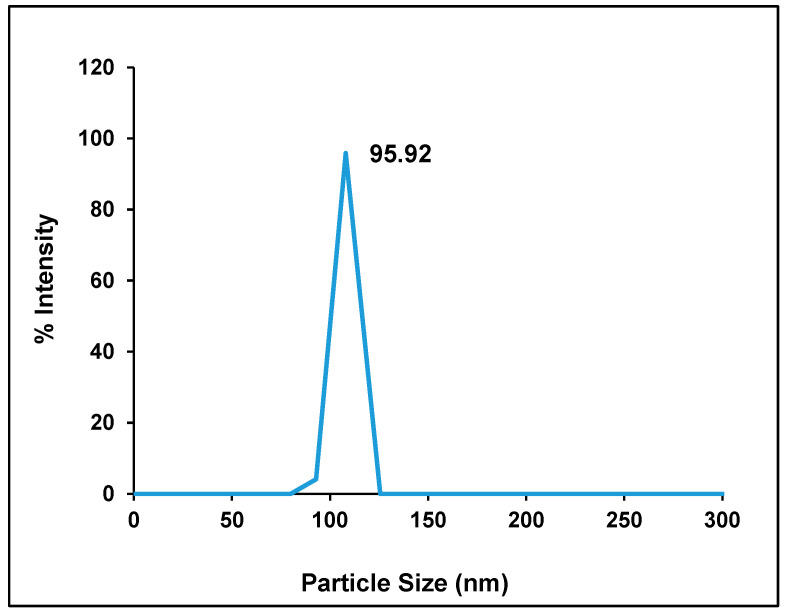
Particle size distribution of Hes-NPs.

Hes-NPs were analyzed using dynamic light scattering (DLS), which revealed particle sizes of less than 200 nm (Figure 1) with a mean value of 127 ± 32.15 nm. The zeta potential average was −51.125 ± 9.79 mV (Table 1). Additionally, chitosan/STPP nanoparticles had an 83% entrapment effectiveness for Hes and a loading capacity (LC) of 31.16%. (Table 1).

### 2.2. Scanning Electron Microscopy (SEM) and Transmission Electron Microscopy (TEM)

**Figure 2:**
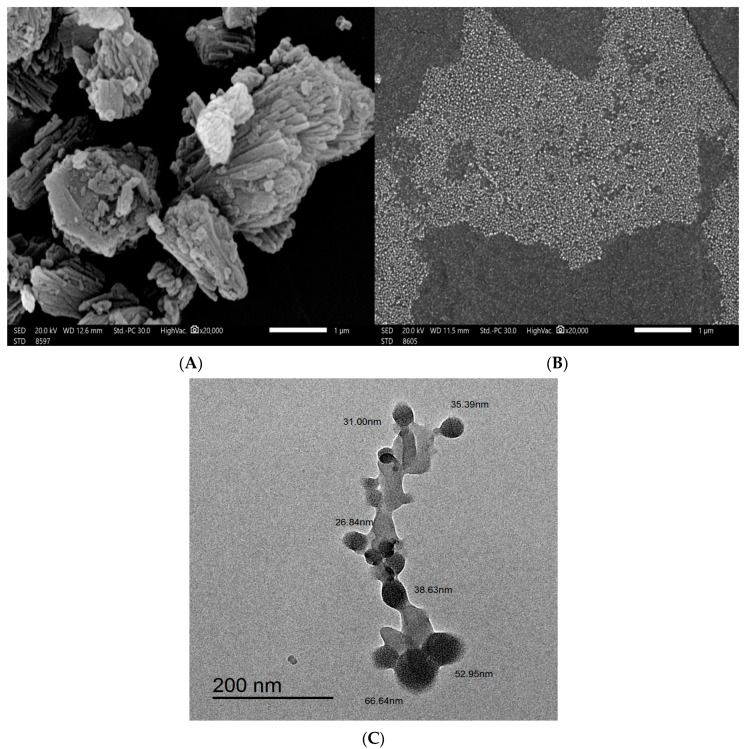
SEM of Hes (**A**) and Hes-NPs (**B**) with magnification power 20,000, and TEM of Hes-NPs (**C**).

Figure 2 depicts how SEM was used to investigate the particle morphology, size, and shape of Hes and Hes-NPs. Figure 2A (magnification power of 20,000) depicts the Hes particles, which were unevenly distributed with variable forms and sizes. The Hes-NPs appear as homogenous spherical clusters (magnification power of 20,000) with essentially equal sizes (Figure 2B).

The cross-linked spherical nanostructure particles with sizes less than 70 nm were easily visible using TEM imaging. The image depicts spherical dark dots that indicate Hes encapsulation within the network (less-shaded area) of chitosan NaTPP (Figure 2C).

### 2.3. X-ray Diffraction Analysis (XRD)

**Figure 3:**
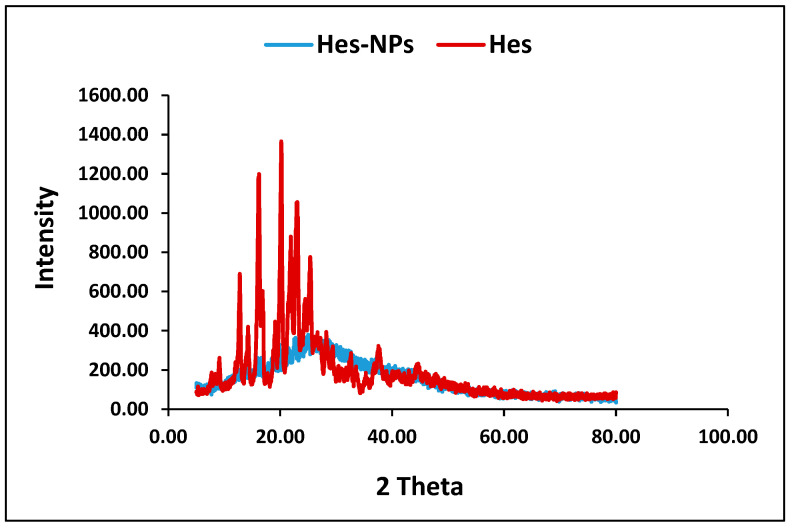
XRD of Hes and Hes-NPs.

The diffractogram of Hes was examined (Figure 3), and characteristic sharp diffraction peaks at 2θ (12.77, 14.24, 16.1, 20.18, 21.89, 22.91, and 25.43°) were identified. On the other hand, the diffractogram of the Hes-NPs shows a much more diminished peak (Figure 3).

### 2.4. FTIR Analysis

**Figure 4:**
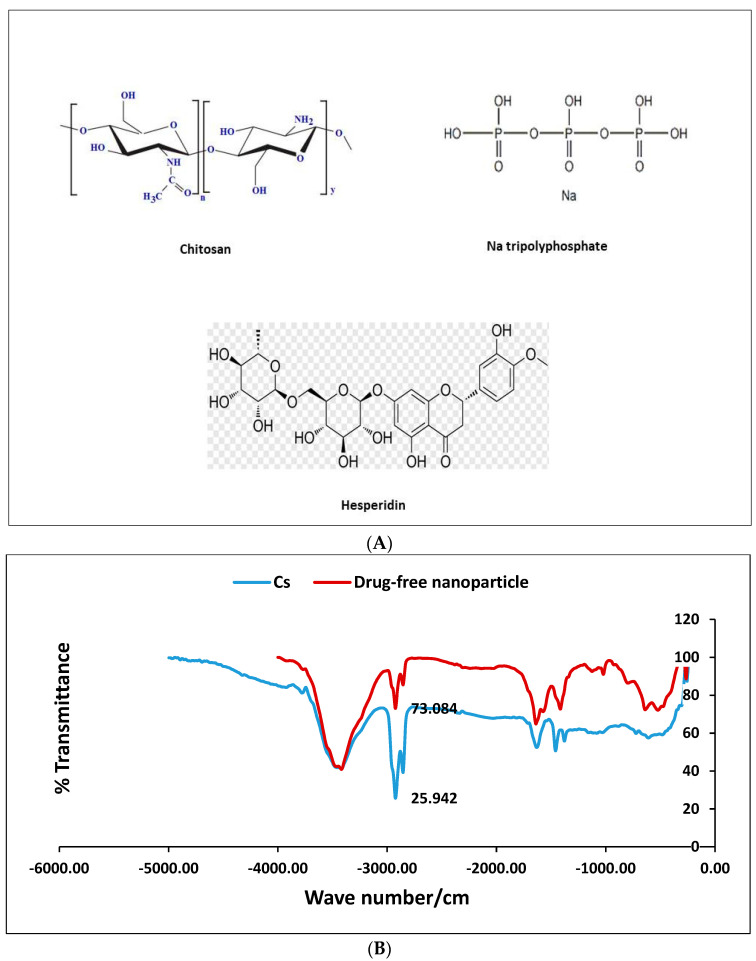

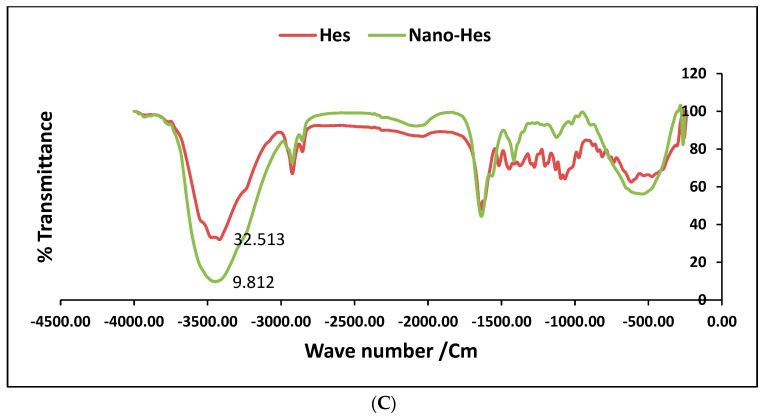
(**A**) The chemical structure of Hes and components of the formulation (Cs and sodium tripolyphosphate). FTIR of drug-free nanoparticles and Cs (**B**) and FTIR of Hes and Hes-NPs (**C**).

The FTIR was conducted for Cs, drug-free nanoparticles, Hes, and nano-Hes. The chemical structure of Hes and the components of the formulation (Cs and sodium tripolyphosphate) are illustrated (Figure 4A). The FTIR of the drug-free nanoparticles was almost the same as the FTIR of the Cs except for the peak at 2925.9/cm, which was related to Sp3 bending, showing a higher transmittance value concerning Cs (Figure 4B). The FTIR of the nano-Hes was almost the same except the peak at 3437.05/cm, showing a lower transmittance than Hes in addition to an increase in the percent transmittance of the fingerprint (Figure 4C).

### 2.5. In Vivo Experiment

#### 2.5.1. Effects of Different Treatments on Kidney Functions

**Figure 5:**
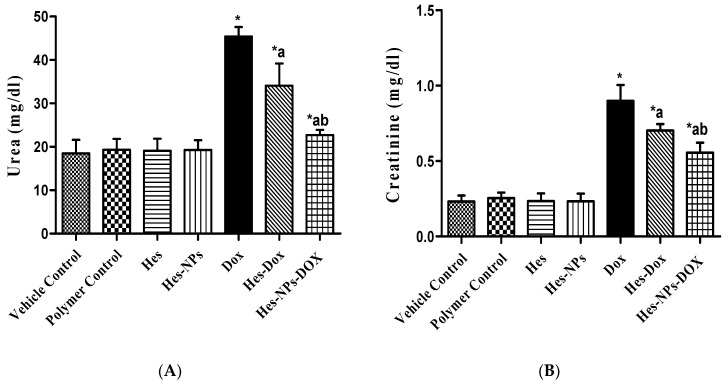
Effect of different treatments on kidney function. Urea (**A**) and creatinine (**B**). Data were expressed as mean ± SD, *n* = 6. * means significant versus vehicle control group, a means significant versus DOX group, and b means significant versus Hes-DOX group. DOX: Doxorubicin, Hes: hesperidin, and Hes-NPs: hesperidin nanoparticles. Each group differed significantly from the others at *p* ≤ 0.05.

In Figure 5, the DOX group shows a notable increase in urea and creatinine serum levels (145.8% and 350%, respectively) compared with those found in the vehicle control group, indicating reduced kidney function. Mice pretreated with Hes demonstrated a decrease in these levels (36.05% and 24.5%, respectively) compared to the DOX group, and a subsequent pretreatment with Hes-NPs induced an even greater decline (50% and 38.88%, respectively). Additionally, the group pretreated with Hes-NPs displayed an extensive decrease in urea and creatinine levels (33.36% and 20.71%, respectively) compared to the Hes group in the presence of DOX.

#### 2.5.2. Effects of Different Treatments on Lipid Peroxidation and Antioxidant Enzyme Activity (CAT and SOD) Estimated in Kidney Tissue

**Figure 6:**
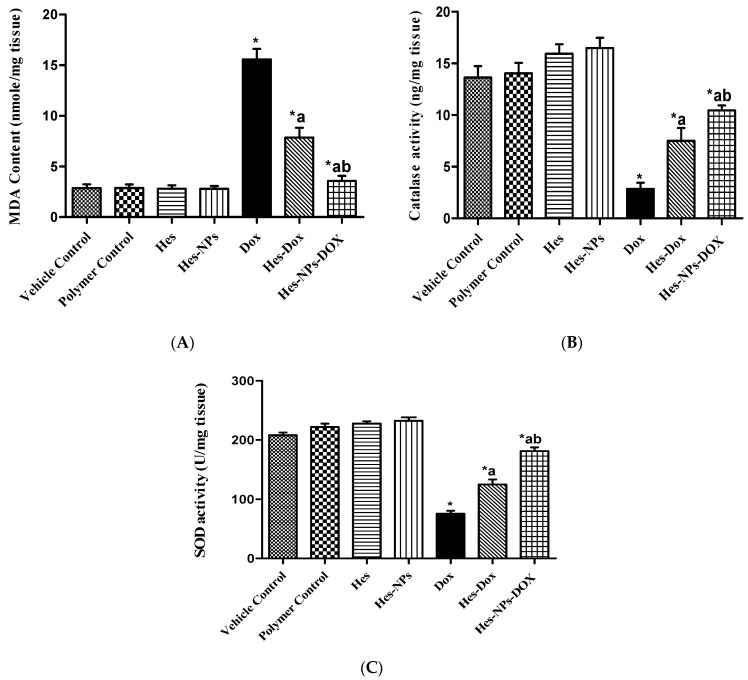
Effect of different treatments on lipid peroxidation and antioxidant enzyme activities: MDA content (**A**), CAT activity (**B**), and SOD activity (**C**). Data were expressed as mean ± SD, *n* = 6. * means significant versus vehicle control group, a means significant versus DOX group, and b means significant versus Hes-DOX group. DOX: Doxorubicin, Hes: hesperidin, and Hes-NPs: hesperidin nanoparticles. Each group differed significantly from the others at *p* ≤ 0.05.

As shown in Figure 6A, the DOX group demonstrated a significant increase in MDA content (444.76%) compared to the vehicle control group. Conversely, groups pretreated with Hes and Hes-NPs showed an extensive reduction (49.55% and 96.41%, respectively) in MDA content in comparison to the DOX group. Furthermore, mice pretreated with Hes-NPs revealed a 54.71% reduction in MDA content in comparison to the Hes group in the presence of DOX (Figure 6A).

The CAT enzyme activity in the DOX group was markedly reduced (79.09%) compared to the vehicle control group (Figure 6B). Additionally, the decrease in CAT kidney activity was reverted by pretreatment with Hes and Hes-NPs (163.50% and 267.02%, respectively) in comparison to the DOX group. Furthermore, pretreatment with Hes-NPs resulted in a remarkable increase (39.28%) in CAT activity in comparison to the Hes group in the presence of DOX (Figure 6B).

The SOD enzyme activity results showed that the DOX group had considerably reduced activity (63.71%) in comparison to the vehicle control group. The exhausted activity was recovered by pretreatment with Hes and Hes-NPs (65.71% and 140.4%, respectively) in comparison to the DOX group. Mice pretreated with Hes-NPs showed a significant increase in enzyme activity (45.06%) compared to the Hes group in the presence of DOX (Figure 6C).

#### 2.5.3. Effect of Different Treatments on Content of Inflammatory Cytokines (TNF-α and IL-1β)

**Figure 7:**
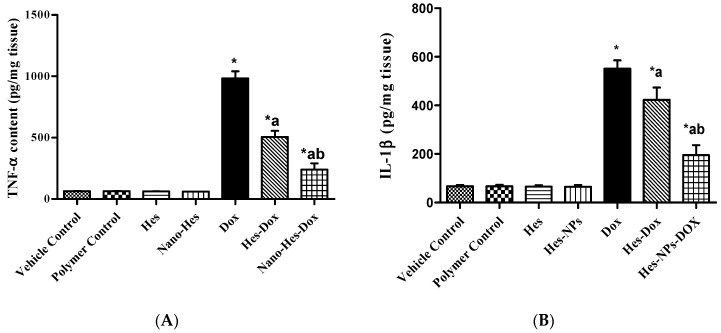
Effect of different treatments on inflammatory cytokine content: TNF-α (**A**) and IL-1β (**B**). Data were expressed as mean ± SD, *n* = 6. * means significant versus vehicle control group, a means significant versus DOX group, and b means significant versus Hes-DOX group. DOX: Doxorubicin, Hes: hesperidin, and Hes-NPs: hesperidin nanoparticles. Each group differed significantly from the others at *p* ≤ 0.05.

As revealed in Figure 7A, the DOX group exhibited a significant increase (684.98%) in TNF-α content compared to the vehicle control group. Conversely, pretreatment with Hes and Hes-NPs elicited an extensive reduction (34.19% and 52.96%, respectively) in TNF-α content in comparison to the DOX group. Furthermore, mice pretreated with Hes-NPs revealed a reduction (29.77%) in TNF-α content compared to the Hes group in the presence of DOX (Figure 7A).

The IL-1β content in the DOX group was markedly reduced (720.86%) in comparison to the vehicle control group (Figure 7B). Additionally, the depletion in IL-1β kidney content was reverted by pretreatment with Hes and Hes-NPs (23.24% and 181.43%, respectively) in comparison to the DOX group. Furthermore, the group pretreated with Hes-NPs showed a remarkable increase (53.72%) in IL-1β content in comparison to the Hes group in the presence of DOX (Figure 7B).

#### 2.5.4. Effect of Different Treatments on VEGF Content

**Figure 8:**
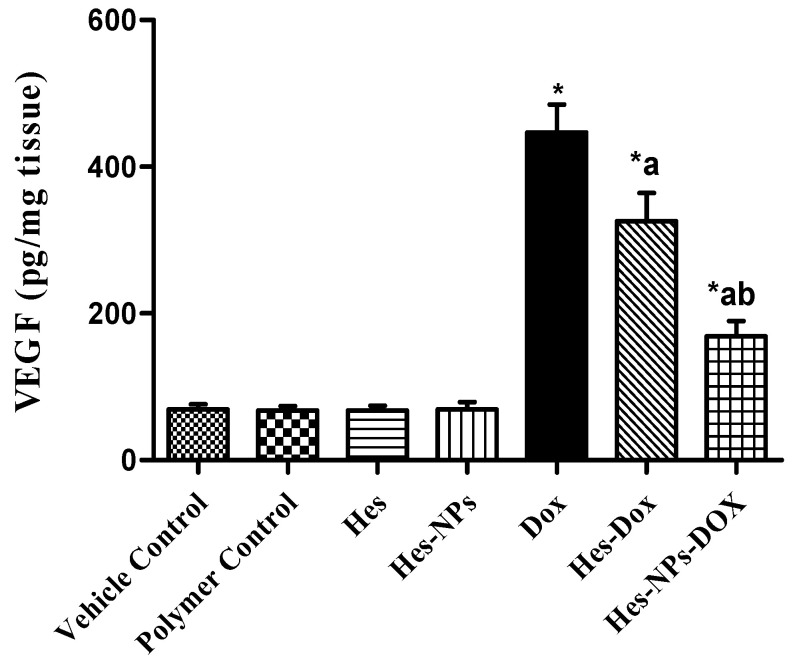
Effect of different treatments on VEGF content. Data were expressed as mean ± SD, *n* = 6. * means significant versus vehicle control group, a means significant versus DOX group, and b means significant versus Hes-DOX group. DOX: Doxorubicin, Hes: hesperidin, and Hes-NPs: hesperidin nanoparticles. Each group differed significantly from the others at *p* ≤ 0.05.

As shown in Figure 8, the DOX group exhibited a notable increase in VEGF content (545.64%) relative to the vehicle control group. Further, groups pretreated with Hes and Hes-NPs demonstrated a remarkable reduction (27.08% and 164.81%, respectively) in VEGF content relative to the DOX group. Moreover, mice pretreated with Hes-NPs displayed a considerable decline (93.09%) in VEGF content in comparison to the Hes group in the presence of DOX (Figure 8).

#### 2.5.5. Effect of Different Treatments on Gene Expression of *Sirt-1, Bcl-2, VEGF, HIF1-α*, and *Kim-1*

**Figure 9:**
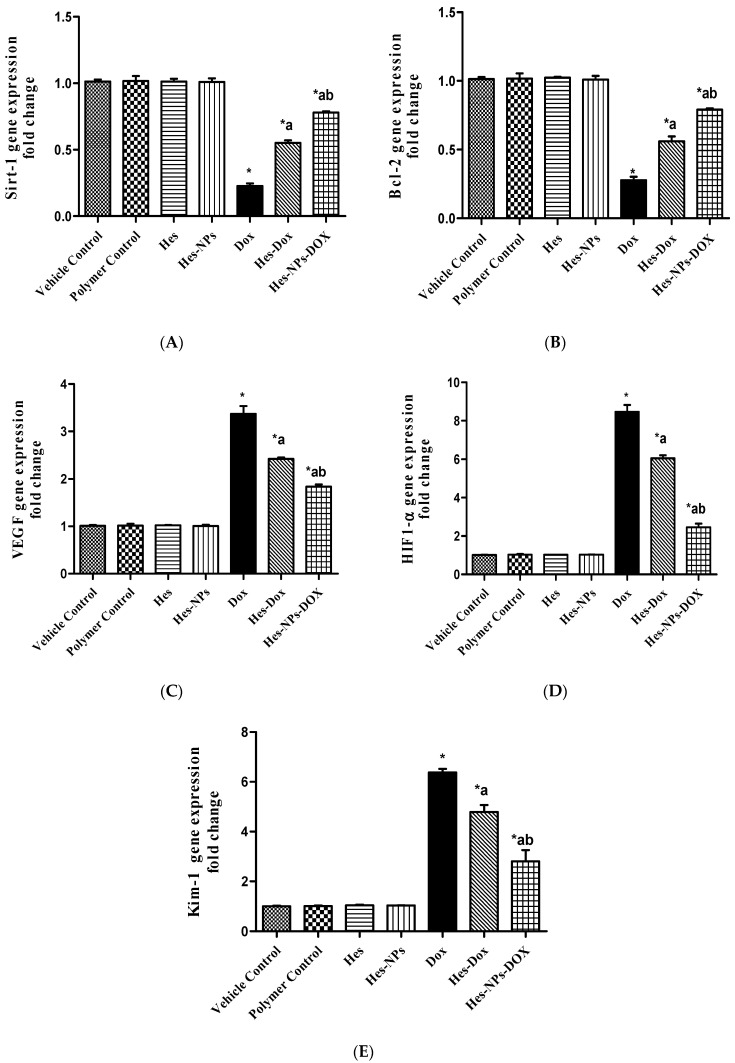
Effect of different treatments on gene expression of *Sirt-1* (**A**), *Bcl-2* (**B**), *VEGF* (**C**), *HIF1-α* (**D**), and *Kim-1* (**E**). Data were expressed as mean ± SD, *n* = 3. * means significant versus vehicle control group, a means significant versus DOX group, and b means significant versus Hes-DOX group. DOX: Doxorubicin, Hes: hesperidin, and Hes-NPs: hesperidin nanoparticles. Each group differed significantly from the others at *p* ≤ 0.05.

As illustrated in Figure 9A, the DOX group displayed a remarkable downregulation (80%) of the kidney’s level of *Sirt-1* gene expression relative to the vehicle control group. However, groups pretreated with Hes and Hes-NPs displayed a substantial upregulation of *Sirt-1* gene expression (143% and 245%, respectively) in comparison to the DOX group. Additionally, the group pretreated with Hes-NPs showed a substantial upregulation (41.81%) of *Sirt-1* gene expression in comparison to the Hes group in the presence of DOX (Figure 9A).

Likewise, the DOX group presented a significant downregulation (72%) of the kidney’s level of *Bcl-2* gene expression relative to the vehicle control group, as shown in Figure 9B. However, groups pretreated with Hes and Hes-NPs displayed a substantial upregulation (147.79% and 182.14%, respectively) of *Bcl-2* gene expression in comparison to the DOX group. Additionally, the group pretreated with Hes-NPs showed a substantial upregulation (41.07%) of *Bcl-2* gene expression in comparison to the Hes group in the presence of DOX (Figure 9B).

On the other hand, as demonstrated in Figure 9C, the DOX group presented a substantial upregulation (237%) in the kidney level of *VEGF* gene expression relative to the vehicle control group, and the groups pretreated with Hes and Hes-NPs displayed substantial downregulation (28.18% and 45.69%, respectively) in this gene expression compared to the DOX group. In addition, the group subjected to Hes-NP pretreatment showed substantial downregulation (24.38%) in *VEGF* gene expression compared to the Hes group in the presence of DOX (Figure 9C).

As presented in Figure 9D, the DOX group showed a substantial upregulation (746%) of the kidney level of *HIF1-α* gene expression relative to the vehicle control group, and the groups pretreated with Hes and Hes-NPs displayed a substantial downregulation (28.45% and 70.92%, respectively) of this expression compared to the DOX group. Additionally, the group subjected to Hes-NP pretreatment showed a substantial downregulation (59.34%) of *HIF1-α* gene expression in comparison to the Hes group in the presence of DOX (Figure 9D).

In the same manner, the DOX group presented a considerable upregulation (537%) of the kidney level of *Kim-1* gene expression relative to the vehicle control group (Figure 9E), and the groups pretreated with Hes and Hes-NPs exhibited a significant downregulation (33.26% and 56.04%, respectively) of this expression compared to the DOX group. Furthermore, the group pretreated with Hes-NPs revealed an extensive downregulation (41.42%) of *Kim-1* gene expression in comparison to the Hes group in the presence of DOX (Figure 9E).

#### 2.5.6. Histopathological Assessment

**Figure 10:**
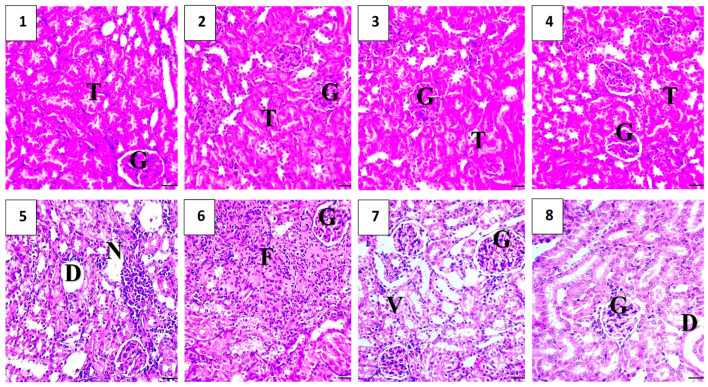
Histopathological evaluation of kidney samples across experimental groups revealed distinct findings. The vehicle control (**1**) and polymer control (**2**) groups exhibited normal histological structures with intact glomeruli, tubules, and interstitium, devoid of inflammation or damage. Hes (**3**) and Hes-NP (**4**) groups preserved kidney architecture similarly to controls, showing only mild microvilli loss. In contrast, the group with DOX-induced renal injury featured severe pathological changes (**5** and **6**), including tubular necrosis, interstitial inflammation, and glomerular congestion. Co-treatment with Hes-DOX (**7**) mitigated these effects, exhibiting moderate histopathological alterations. The Hes-NPs-DOX group (**8**) displayed nearly normal renal cortex architecture. The grading system for evaluating tubular necrosis, loss of brush border, cast formation, and tubular dilatation was applied to 10 randomly selected, non-overlapping fields at 200× magnification. G: Glomeruli, T: tubule, V: vacuolization, D: dilation, F: fibrotic reaction, and N: inflammation. DOX: Doxorubicin, Hes: hesperidin, and Hes-NPs: hesperidin nanoparticles.

As shown in Figure 10, a histopathological evaluation of the kidneys in the vehicle control group revealed a normal histological structure, with the glomeruli, tubules, and interstitium intact and no signs of inflammation or damage. Similarly, the polymer control group exhibited normal kidney histology, with intact Bowman’s capsules and normal proximal and distal convoluted tubules. The Hes-only group showed normal kidney histology, indicating that the hesperidin treatment did not cause significant changes. The Hes-NP group also demonstrated normal kidney architecture, comparable to the control groups. In all four groups, the only histopathological observation was a mild, occasional loss of microvilli.

Conversely, the DOX-damaged group showed significant renal injury upon histopathological analysis. Observations included changes in the endothelial cell cytoplasm of proximal tubules, degeneration or loss of microvilli, disintegration of renal tubules with exfoliated cells, luminal shedding of epithelial cells, cystic dilatation, tubular necrosis, interstitial inflammation, and glomerular congestion, all indicative of doxorubicin-induced renal damage (Figure 10).

The Hes-DOX group exhibited well-preserved cellular and tubular structures in the kidneys, with a loss of microvilli and mild degeneration and dilation, but no necrosis or inflammatory cells were detected. The Hes-NPs-DOX group showed near-normal architecture of the renal cortex. The glomeruli were surrounded by clear Bowman’s spaces, and the proximal and distal tubules appeared largely normal, although some mild dilation and degenerated vacuoles were observed in the lining epithelia of a few tubules (Figure 10).

#### 2.5.7. Immunohistochemical Assessment

**Figure 11:**
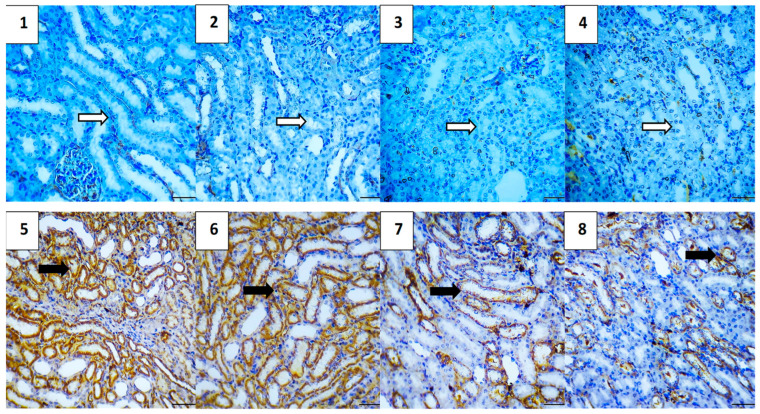
Photomicrographs showing Caspase-3 immunoreactivity (200× magnification). Vehicle control (**1**) and polymer control (**2**) groups showed minimal to no Caspase-3 expression, indicating baseline levels of apoptosis unaffected by these agents. Hes (**3**) and Hes-NP (**4**) groups exhibited negligible Caspase-3 levels, suggesting no induction of apoptosis. Conversely, DOX-treated kidneys showed elevated Caspase-3 expression, indicating increased apoptotic activity (**5**,**6**). Hes-DOX (**7**) and Hes-NPs-DOX (**8**) groups resulted in moderate to minimal Caspase-3 expression. Black arrows indicate positive immunoreaction, while white arrows indicate negative immunoreaction. DOX: Doxorubicin, Hes: hesperidin, and Hes-NPs: hesperidin nanoparticles.

**Figure 12:**
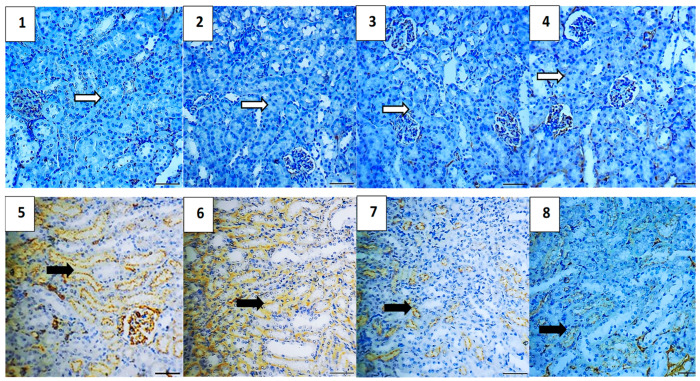
Photomicrographs showing NF-κB immunoreactivity (200× magnification). Vehicle control (**1**), polymer control (**2**), Hes (**3**), and Hes-NP (**4**) groups showed minimal NF-κB immunoreactivity, indicating low inflammation levels under normal conditions. In contrast, DOX-induced injury significantly increased NF-κB expression, highlighting intense inflammatory activity (**5**,**6**). Hes-DOX (**7**) and Hes-NPs-DOX (**8**) groups exhibited reduced NF-κB expression, suggesting potent anti-inflammatory effects. Black arrows indicate positive immunoreaction, while white arrows indicate negative immunoreaction. DOX: Doxorubicin, Hes: hesperidin, and Hes-NPs: hesperidin nanoparticles.

**Figure 13:**
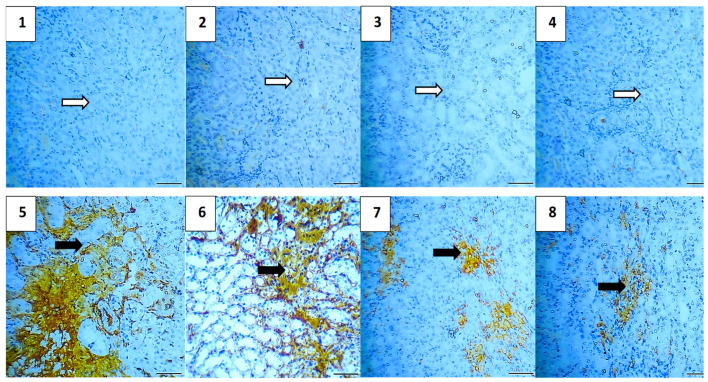
Photomicrographs showing TGF-β1 immunoreactivity (200× magnification). Vehicle control (**1**), polymer control (**2**), Hes (**3**), and Hes-NP (**4**) groups displayed insignificant expression of TGF-β1, indicating minimal fibrotic activity in normal kidneys. Conversely, DOX-induced injury led to significant TGF-β1 alterations, indicative of fibrotic changes (**5**,**6**). Hes-DOX (**7**) and Hes-NPs-DOX (**8**) groups showed minimal TGF-β1 expression, suggesting mitigation of fibrotic pathways by these treatments. Black arrows indicate positive immunoreaction, while white arrows indicate negative immunoreaction. DOX: Doxorubicin, Hes: hesperidin, and Hes-NPs: hesperidin nanoparticles.

**Figure 14:**
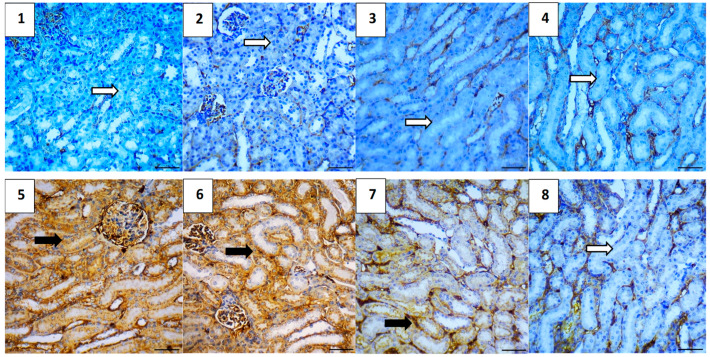
Photomicrographs showing BAX immunoreactivity (200× magnification). Vehicle control (**1**), polymer control (**2**), Hes (**3**), and Hes-NP (**4**) groups showed minimal BAX expression, indicating low pro-apoptotic activity under normal kidney conditions. Conversely, DOX-induced injury led to a significant increase in BAX expression (**5**,**6**), suggesting severe induction of apoptotic pathways. Hes-DOX (**7**) and Hes-NPs-DOX (**8**) groups attenuated an increase in BAX expression, indicating partial inhibition of doxorubicin-induced apoptosis, with a greater reduction in BAX expression in the Hes-NPs-DOX group. Black arrows indicate positive immunoreaction, while white arrows indicate negative immunoreaction. DOX: Doxorubicin, Hes: hesperidin, and Hes-NPs: hesperidin nanoparticles.

**Figure 15:**
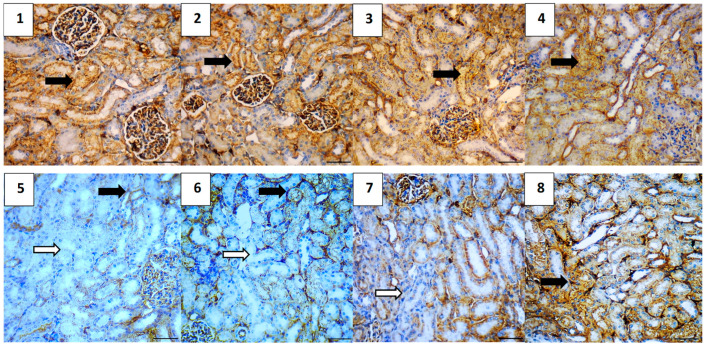
Photomicrographs showing Bcl-2 immunoreactivity (200× magnification). Vehicle control (**1**), polymer control (**2**), Hes (**3**), and Hes-NP (**4**) groups displayed significant Bcl-2 expression levels, indicative of a balanced state between pro-survival and pro-apoptotic signals in normal kidneys. Conversely, DOX-induced injury resulted in decreased Bcl-2 expression, disrupting this balance (**5**,**6**). Hes-DOX (**7**) and Hes-NPs-DOX (**8**) groups maintained mild to moderate Bcl-2 expression. Black arrows indicate positive immunoreaction, while white arrows indicate negative immunoreaction. DOX: Doxorubicin, Hes: hesperidin, and Hes-NPs: hesperidin nanoparticles.

The immunohistochemical analysis provided insights into the expression levels of various markers associated with apoptosis, inflammation, and cell survival across different experimental groups. The vehicle control group showed no expression of Caspase-3 and NF-κB, very low expression of TGF-β1 (0.4) and BAX (0.4), and high expression of Bcl-2 (5.2), indicating a lack of apoptosis and inflammation, and preserved cell survival. Similarly, the polymer control group had low expressions of Caspase-3 and TGF-β1 (0.4 each), no NF-κB expression, low BAX expression (0.4), and high Bcl-2 expression (4.8), suggesting minimal apoptosis and inflammation, and good cell survival (Figure 11, Figure 12, Figure 13, Figure 14 and Figure 15).

In the Hes group, Caspase-3 and NF-κB expressions were slightly elevated (0.4 each), indicating a mild induction of apoptosis and inflammation, while TGF-β1 was absent, BAX was low (0.4), and Bcl-2 remained high (5.0), indicating strong cell survival. The Hes-NP group showed similar trends with low expressions of Caspase-3 and NF-κB (0.4 each), absent TGF-β1, low BAX (0.4), and high Bcl-2 (5.0), suggesting slight apoptosis and inflammation but strong cell survival (Figure 11, Figure 12, Figure 13, Figure 14 and Figure 15).

The DOX-damaged group exhibited high expression levels of Caspase-3 (4.6), NF-κB (4.4), TGF-β1 (3.8), and BAX (5.0), reflecting high levels of apoptosis, inflammation, and tissue damage. It also showed a lower Bcl-2 expression (2.5), indicating reduced cell survival. The Hes-DOX group showed moderate protection with Caspase-3 (3.6), NF-κB (2.6), TGF-β1 (2.0), BAX (3.2), and Bcl-2 (3.4), indicating balanced apoptosis and cell survival. The Hes-NPs-DOX group demonstrated the best protection, with lower expressions of Caspase-3 (2.4) and NF-κB (1.6), reduced apoptosis and inflammation, low TGF-β1 (1.2), moderate BAX (2.0), and relatively high Bcl-2 (4.4), indicating better cell survival (Figure 11, Figure 12, Figure 13, Figure 14 and Figure 15).

**Figure 16:**
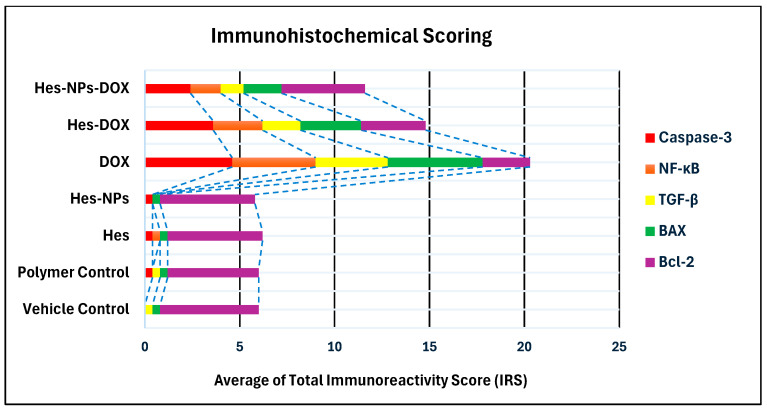
Average total immunoreactivity scores (IRSs) for various markers across experimental groups.

Immunohistochemical findings were evaluated based on the staining intensity and the extent of the stained area. The total immunoreactivity score (IRS) for each tissue section was calculated by summing the intensity and area scores (Table 2 and Figure 16).

## 3. Discussion

The biological effects of citrus flavonoids such as hesperidin (Hes) are diverse. They have been shown to have antioxidant, anti-inflammatory, and anti-apoptotic activities [24]. About 20% of Hes molecules are bioavailable [29,30]. Only a modest number of Hes molecules are released into the aqueous environment of the gastrointestinal system due to its poor water solubility [30]. Hence, new dosage formulations are required to increase its therapeutic effectiveness. Because of glomerular filtration barrier size selectivity, NP size has a significant impact on biodistribution and therapeutic potential [31]. The termed “nanoparticles” are increasingly popular for their use as drug delivery systems to overcome classical problems faced by most drugs such as low solubility, low bioavailability, non-specificity, and/or toxicity [32]. Nanoparticle dimensions have been reported to influence the ability to bind and diffuse through mucus, as the mucus’ mesh pore size (10–200 nm) sterically limits nanoparticles larger than 200 nm [33].

Chitosan is a readily available biomaterial, and numerous synthesis methods have been tested to produce chitosan nanoparticles [33,34]. A modified ionic gelation method was conducted with the aid of an ice bath. The ice bath is composed of Oasis floral foam which was specially adapted to incubate the reaction bottle. The Oasis foam is soaked with water and then transferred into an ultra-low freezer (BINDER GmbH, Tuttlingen, Germany) for 24 h (−80 °C) before the experiment. The ice bath helps to slow down the process of ionic gelation so it can enable the formation of relatively small particles. On the other hand, it helps to reduce the heat generated during the utilization of a probe sonicator [35]. The particle size was assessed through TEM, SEM, and DLS; the TEM examination illustrated the encapsulation of Hes, where, inside the chitosan polymer, the particle size was less than 100 nm and the shape was detected as spherical capsules embedded in the chitosan network.

Dynamic light scattering was utilized to measure the hydrodynamic diameter of dispersed particles and is used as a quick guide for the success of particle size reduction at the beginning of the preparation. DLS measured the hydrodynamic diameter with a mean value of 127 ± 32.15 nm.

The SEM technique determined both the shape and size of the nanoparticles in comparison with the hesperidin powder. On the other hand, the TEM technique gave a true shape and actual particle size rather than the hydrodynamic diameter determined by dynamic light scattering and is considered the best method for particle-size analysis, especially for chitosan, due to its ability to swell in aqueous solution, in addition to its exact shape determination. Moreover, TEM imaging illustrated the intermolecular and intramolecular hydrogen bonds within chitosan polymer, giving the shape of a network of polymer-entrapping spherical particles (dark spheres) encapsulating Hes. Finally, the XRD analysis gave information about particle size and the degree of crystallinity (both affecting the dissolution and hence the absorption following oral administration). The XRD analysis of Hes-NPs showed hump-like diffraction with the disappearance of the characteristic peaks of Hes; these findings supported the transformation of Hes into amorphous nano-sized particles [36].

The FTIR of chitosan was characterized by a broad strong band at 3421/cm, which was related to N-H and O-H stretching in addition to its intermolecular hydrogen bonds. There was also C-H symmetric and asymmetric stretching at 2925.9 and 2854.5/cm, respectively, and the band of C=O stretching of the amide group was at 1625/cm and C-N stretching of the amide group at 1380/cm [37].

The FTIR of drug-free nanoparticles had almost the same bands, except the % transmittance was greater in all bands except the bands of N-H and O-H stretching, which meant the formation of hydrogen bonds occurred to the same extent for the polymer and drug-free nanoparticles. The FTIR spectra of Hes revealed distinct bands due to the presence of various functional groups, including O-H stretching vibration (3415.8/cm), C-H stretching (2924/cm), C=O stretching (1643/cm), C=C stretching, and C-O stretching (1070/cm). The later bands in the FTIR spectrum are due to the in-plane and out-plane bending modes present in Hes [38]. The FTIR of the Hes-NPs was almost the same, except it contained a lower % transmittance of the band corresponding to O-H stretching vibration, which can be explained by a chemical interaction between Hes and chitosan polymer with a possible hydrogen bond between Hes and Cs. In addition, there was an increase in the % transmittance in the fingerprint region, which can be attributed to the encapsulation of Hes inside the polymer matrix. The encapsulation of Hes in chitosan utilizing the ionic gelation method showed an improved dissolution behavior in 0.1 N HCl [10].

Doxorubicin (DOX) is a chemotherapeutic drug used to manage certain malignancies, such as stomach, lung, breast, ovarian, and pediatric cancers [39]. However, continued usage of DOX can severely impact the body’s organs, such as the heart, liver, and kidney [40]. Such side effects limit the clinical application of DOX despite its therapeutic efficacy. As the kidney is the main executive organ in the human body, its injury due to the toxicity of DOX is a major clinical problem that has been widely studied. The nephrotoxicity triggered by DOX usually occurs because of the development of various free radicals, which induce oxidative damage [41].

At low concentrations, reactive oxygen species (ROS), which are partly reduced metabolites of oxygen with potent oxidizing properties, perform intricate signaling roles within cells. At large concentrations, ROS are harmful to cells. Reactive oxygen species are necessary for cellular signaling and homeostasis maintenance since they are produced as byproducts of regular cell metabolism. In addition to being produced by certain plasma membrane oxidases in response to cytokines and growth factors, they also function as secondary messengers in particular signaling pathways and are involved in the regulation of gene expression [42]. Cells have a defense system to maintain ROS at physiologically normal levels, i.e., enzymes called antioxidants, responsible for transforming free radicals into stable, less damaging molecules, the impairment of which may lead to a state of oxidative stress [43]. These oxygen-scavenging pathways include the conversion of O_2_− to H_2_O_2_ by superoxide dismutase (SOD), the action of catalase on H_2_O_2_ to produce H_2_O and O_2_, the decomposition of H_2_O_2_ and LOOH by glutathione peroxidase, and the reduction of H_2_O_2_ through the thioredoxin reduction cycle to produce H_2_O, and also the exogenous detoxification of glutathione transferase [44].

Cancer cells have a high metabolic activity and are hypoxic. As a result of their rapid growth and inadequate vascular irrigation, they tend to produce more reactive oxygen species (ROS), which can damage DNA by permeating the mitochondrial membrane and acting as signaling molecules in a variety of redox-sensitive molecular pathways that are important for cell survival, treatment resistance, and progression [44].

Since oxidative stress is linked to several cancer hallmarks, including angiogenesis, invasiveness, stemness, and metastatic potential, lowering oxidative stress through the use of potent antioxidants has been a key component of cancer-prevention research [45,46,47]. Furthermore, cancer cells create defenses against elevated oxidative stress. Because of this, several cancer-treatment approaches also function by interfering with this check and rendering cancer cells vulnerable to death [48]. Additionally, it has been reported that DOX initiates the inflammatory process by activating the NF-κB pathway [49]. Furthermore, it was reported that DOX can trigger apoptosis by stimulating the proteolytic processing of the Bcl-2 family and caspases [50].

Antioxidants play a role in adjuvant chemotherapy because they react to and eliminate oxidizing free radicals, which prevent cellular damage. According to [51], between 13 and 87% of cancer patients use antioxidant supplements. However, in cancer treatment, certain antineoplastic drugs work by generating free radicals, which further damage cells and cause malignant cells to necrotize [52]. Antioxidant usage during chemotherapy is thus discouraged for fear that it will interfere with the medication’s effectiveness. Conversely, a lot of integrative practitioners discuss the usage of antioxidant supplements, which enable patients to withstand potentially more potent chemotherapy dosages, improving the chance of a stronger tumor response and a greater survival rate [52].

Antioxidant-active herbal substances have drawn a lot of interest in the realm of cancer treatment [53]. It has been noted that natural substances found in food, particularly bioactive substances called flavonoids, can prevent cancer from developing and help treat cancer [53]. Apart from being anti-inflammatory and antioxidant, flavonoids have antihypertensive and antiallergic activity, and they disrupt the three phases of carcinogenesis as well [54,55]. The most prevalent flavonoid in citrus fruits is hesperidin, which is a flavanone glycoside that is non-toxic and non-allergic and has no negative side effects [54,55]. Recently, the pharmacological and biological effects of hesperidin have been investigated [56,57]. Several investigations have proposed its anti-cancer properties through suppressing tumor growth and proliferating and promoting programmed cellular death (e.g., in colon, breast, and prostate cancer cells) [57,58,59]. Another recent study strongly implies that hesperidin may have a synergistic effect that may be harnessed to improve DOX’s anticancer efficacy and lower the hazards associated with using chemotherapy for metastatic breast cancer [60].

In the current study, we aimed to use Hes-NPs to overcome the nephrotoxic side effects of DOX based on the previously reported antioxidant and anti-inflammatory potentials of Hes [61,62]. The nanoformulation of Hes was used in the current study to improve its dissolution rate, as its insolubility in water significantly restricts its clinical usefulness.

The deterioration of kidney functions is a hallmark of nephrotoxicity. It presents a rise in urea serum, creatinine serum, and blood urea nitrogen (BUN) [63]. In this study, there was a remarkable increase in such indices in the DOX-treated mice, indicating the DOX-induced loss of renal functions. Moreover, the gene expression of KIM-1, a nephrotoxic biomarker [64], was increased, indicating renal impairment. All these indices were substantially decreased in the Hes-treated group, consistent with previous studies [65,66]. In addition, this effect was more promising in the nano-Hes-treated group, which could be attributed to the increased bioavailability of the prepared nanoformulation.

The renal-protective action of Hes-NPs was further confirmed using histopathological studies with H&E staining, as there was a decrease in the kidney infiltration of inflammatory cells in the nano-Hes-treated group. Additionally, the histopathologic examination of the DOX-treated group revealed noticeable pathological lesions in the kidney tissues and a worsening in the kidney architecture, which agrees with previous studies [67,68]. This was remarkably improved in the Hes-NP-treated group.

As shown in previous studies [69,70], DOX considerably reduced the activity of antioxidant enzymes, including SOD and CAT, accompanied by a significant rise in the ROS level and lipid peroxidation, manifested by the MDA level. Lipid peroxidation is a major mark of oxidative stress and can initiate irreversible cell membrane damage [71]. Interestingly, the group treated with Hes-NPs revealed a significant increase in the antioxidant enzyme activity with a remarkable decrease in the ROS and MDA levels. Hes was reported in previous studies to alleviate oxidative stress [72,73], and here, the prepared nanoformulation boosted this effect. Oxidative stress usually arises if there is an imbalance between the oxidant and antioxidant mediators [74].

Sirt1 is a nicotinamide adenine dinucleotide-dependent deacetylase that can regulate oxidative stress, apoptosis, and inflammation in cells [75]. Its significant role in oxidative metabolism was elucidated by inducing certain oxidative stress defense markers, such as CAT and SOD [76]. Thus, the upregulation of the gene encoding *Sirt1* could safeguard the cell from the damage triggered by oxidative stress [77]. In the current study, qRT-PCR showed that Hes-NPs exhibited an upregulation effect on the *Sirt1* gene. The renal-protective influence of the nano-Hes could be partially explained by the mitigation of the oxidative stress induced by DOX via upregulating Sirt1 and the consequent increase in antioxidant enzyme activity. Other researchers observed such outcomes in previous investigations after treatment with Hes [78,79], and the nanoformulation substantially enhanced this effect in our study.

As previously mentioned, the renal-damaging effect of DOX is attributed to its ability to induce oxidative stress, inflammation, and apoptosis. Although oxidative stress is a major mechanism of this damage, the inflammatory response and apoptosis induced by DOX also contribute to renal toxicity [80].

Inflammation is a host response triggered by various contributors, such as invading pathogens and tissue injury [81]. A modest inflammatory response is beneficial and helps re-establish affected tissues [82]. However, a prolonged inflammatory process can lead to increasing damage to the tissues. The IL-1β cytokine is considered a main mediator of inflammation, as it is vital in activating the production of many other inflammatory cytokines [83]. DOX can trigger inflammation in the renal tissues [84], and this was observed in the current study by measuring the substantial rise in the levels of proinflammatory cytokines such as IL-1β and TNF-α.

It has been reported that Sirt1 controls various cellular processes via its deacetylation potential of different transcription factors, such as P53 and NF-κB [85]. Earlier studies have documented the suppressing action of Sirt1 on NF-κB, a key regulator of many proinflammatory cytokines [86,87]. Furthermore, Sirt1 was reported to regulate the P53 signaling pathway, which is involved in the apoptosis process [88]. Thus, targeting Sirt1 could be a beneficial therapeutic approach in controlling DOX-induced renal toxicity by lessening oxidative stress, inflammation, and apoptosis. In agreement with previous studies [89,90], we found that DOX downregulated Sirt1 gene expression, an effect that was significantly alleviated by Hes-NP treatment. The immunohistochemistry studies revealed a substantial rise in NF-κB, which would trigger inflammation in the DOX-treated group in accordance with previous studies [91,92]. Hes decreased NF-κB and its downstream inflammatory cytokines (IL-1β and TNF-α), and this effect was significantly augmented by its formulation as Hes-NPs.

Apoptosis (programmed cell death) is crucial in removing damaged and old cells. The Bcl-2 family comprises proteins that can regulate the process of apoptosis. It includes promoters of cell death, such as BAX, and inhibitors of cell death, such as Bcl-2 [93]. It was reported that an elevated BAX/Bcl-2 ratio is usually connected to an increased vulnerability to the activation of apoptosis [94]. In our investigation, DOX provoked apoptosis in the renal tissue by increasing BAX and decreasing Bcl-2, as revealed by the performed immunohistochemical studies, a finding that agrees with previous research [95,96]. Remarkably, Hes-NPs were found to reduce BAX and increase Bcl-2. Thus, Hes-NPs could have an anti-apoptotic influence by controlling the Bcl-2 family of proteins. These findings agree with other studies documenting the anti-apoptotic impact of Hes [97,98,99] and were amplified in the current investigation by the prepared nanoformulation.

Along with the Bcl-2 family of proteins, a group of cysteine-type proteases (caspases) has been found to have a significant role in apoptosis [100]. These enzymes are synthesized as inactive proenzymes and then processed in the cells to undergo apoptosis. Caspase-3, in particular, is a crucial protease-activated enzyme in the apoptotic process [101]. In this study, DOX significantly increased the level of Caspase-3, and this was alleviated by nano-Hes, which confirms its anti-apoptotic impact.

HIF-1α is a protein that plays a vital role in the body’s response to hypoxia by increasing vascularization. In the inflammatory process, HIF-1α can be induced via oxygen-independent mechanisms mediated by transcription factors such as STAT3 and NF-κB [102]. There is intimate bidirectional crosstalk between NF-κB and HIF-1α. This is due to the reported induction of HIF-1α by NF-κB and the regulatory potential of HIF-1α toward NF-κB [103]. Additionally, it was reported that the level of HIF-1α was found to be higher in the inflammatory cells from wounds, and this was attributed to the upregulation of HIF-1α by the proinflammatory cytokines TNF-α and IL-1β via the NF-κB/COX-2 pathway [104]. In the current study, DOX was found to increase the immune expression of NF-κB and upregulate HIF-1α. On the other hand, such effects were reversed by treatment with Hes-NPs. Many studies have reported the inhibitory effect of Hes on HIF-1α [105,106].

HIF-1α and VEGF are vital regulators of the process of angiogenesis [107]. HIF-1α can activate the transcription of genes that encode glycolytic enzymes, glucose transporters, and VEGF [108]. VEGF is known as a mediator of angiogenesis via its ability to increase microvascular permeability. This is why VEGF is usually upregulated in angiogenic ailments, such as inflammatory reactions, atherosclerosis, liver injury, and kidney diseases [109]. Therefore, the *VEGF* expression level could be utilized as a biomarker for these disorders [110]. In this study, *VEGF* was upregulated in the DOX-treated group and downregulated in the Hes-NP-treated group. This is in accordance with previous studies which documented the inhibitory effect of Hes on *VEGF* [111,112].

TGF-β has been documented to be a main mediator of renal fibrosis and can be produced by various cells, such as macrophages, T lymphocytes, and renal cells [113]. In the current study, nano-Hes remarkably diminished the TGF-β_1_ level. *TGF-β_1_* expression in the kidney tissues is regarded as a final pathway predisposing to fibrosis and structural damage [114,115]. *TGF-β1* could be used as a biomarker for the severity of the glomerular injury [116]. In the current investigation, renal TGF-β_1_ levels were substantially increased in the DOX group, and treatment with nano-Hes subsequently lessened this issue. Previous studies have reported the diminishing potential of Hes on TGF-β_1_ levels [117].

Many biomarkers indicate renal damage, but they are non-specific. Thus, it is essential to discover new biomarkers with high specificity and sensitivity [118]. It was reported that KIM-1 is a very specific urinary biomarker for detecting renal injury by various causes, such as DOX exposure [118]. This study found that DOX increased the gene expression of KIM-1, indicating kidney injury, which was remarkably reduced after treatment with Hes-NPs.

## 4. Materials and Methods

### 4.1. Drugs and Chemicals

Low-molecular-weight chitosan was purchased from Sisco Research Laboratory, India. Sodium tripolyphosphate (85%) was obtained from Lanxess Company, Nagda, Madhya Pradesh, India. El Nasr Pharmaceutical Chemicals Co., Cairo, Egypt, provided high-quality sodium hydroxide (NaOH) and glycerin. Acetic acid (96%) was bought from Research-Lab Fine Chem Industries, Mumbai, India. Deionized water was purchased from Stakpure Waters, Milford, MA, USA. Hesperidin, dimethyl sulfoxide (DMSO), and polyethylene glycol (PEG) were procured from Sigma-Aldrich, Saint Louis, MO, USA. Doxorubicin (Adricin®) was obtained from Hikma Pharmaceuticals, Cairo, Egypt. For every chemical employed in this investigation, the highest analytical grade was utilized.

### 4.2. The Ionic Gelation Method Used for the Preparation of Hesperidin Nanoparticles (Hes-NPs)

Chitosan (Cs) nanoparticles cross-linked with sodium tripolyphosphate (NaTPP) were synthesized using a modified ionotropic gelation process [119].

Chitosan was dissolved in 1% acetic acid (to prepare a 2% Cs solution) with the aid of a magnetic stirrer (Stuart, Calibre Scientific, MI, USA) at 200 rpm and 50 °C for 30 min. One gram of hesperidin was triturated with 1 ml glycerin to form a smooth paste, then 10 mL 1% tween solution was added and disseminated with the aid of a probe sonicator (Sonic Vibra Cell, Newtown, CT, USA) for 5 min in an ice bath (10 s pulse and 5 s pause) at 65% power (130 W).

The homogeneously dispersed Hes was added gradually to the chitosan solution and agitated for an additional 10 min. The dispersion was then agitated for the second time with the probe sonicator (Sonic Vibra Cell, Newtown, CT, USA) for an additional 5 min in an ice bath (10 s pulse and 5 s break) at 65% of its power (130 W), then the pH was adjusted to 5 (with the aid of 4% NaOH). The mixture was incubated in an ice bath (−4 °C). The estimated volume of 4 mL NaTPP 2.5% (*w*/*v*) was added dropwise with a 20 mL syringe, and after 30 min of stirring, the dispersion was returned to the probe sonicator (Sonic Vibra Cell, Newtown, CT, USA). Finally, the nanoparticles were separated with a cooling centrifuge set to 10,000 rpm for 10 min at −40 degrees Celsius (Centurion Scientific, Wolflabs, Pocklington, UK). The nanoparticles were then rinsed twice with deionized water and freeze-dried until totally dry (Christ Benchtop Freeze Dryer, Osterode am Harz, Germany).

The entrapment efficiency was determined using a direct method in triplicate. Five milligrams of freeze-dried nanoparticles were dispersed in a 1% acetic acid solution and transferred into a 25 mL volumetric flask. The Hes concentration was measured using the spectrophotometric method at a wavelength of 285 nm [119].

The loading capacity was calculated by dividing the weight of the known amount of encapsulated medication by the weight of the entire nanoparticle sample.

### 4.3. Characterization of Hesperidin Nanoparticles (Hes-NPs)

#### 4.3.1. Particle Size, Zeta Potential, and Entrapment Efficiency of Hes-NPs

Dynamic light scattering was utilized to estimate the particle size of the colloidal dispersion, while zeta potential evaluated colloidal stability and homogeneity, respectively. A Zetasizer Nano (Malvern Analytical Ltd., Malvern, UK) was used for measuring particle size and zeta potential. A few particles were suspended in deionized water at ambient temperature (samples were allowed to equilibrate for 5 min).

#### 4.3.2. Scanning Electron Microscopy (SEM) and Transmission Electron Microscopy (TEM)

Scanning electron microscopy (SEM) was used to investigate the structure and surface properties of Hes-NPs and Hes. After sonicating and suspending the lyophilized powder in alcohol, one drop of the suspension was spread on a glass slide and allowed to dry fully before being applied to the top of a metal stub (cupper) on a silicon electro-conductive chip. The materials were coated with gold for one minute on the stubs before being examined at various magnifications using a 10 kV electron acceleration voltage field-emission scanning electron microscope (JEOL, JSM-6510LV, Tokyo, Japan). The samples were examined at a magnification power of 20,000.

The nanoparticles were suspended in ethyl alcohol, then put on a carbon grid and dried. The sample was observed and photographed using a transmission electron microscope (TEM, JEM2100F electron microscope, JEOL, Ltd., Tokyo, Japan).

#### 4.3.3. X-ray Diffraction Analysis (XRD) and FTIR

Hes and Hes-NPs underwent XRD analysis. The X-ray diffractograms based on Bragg’s law were acquired using an XRD diffractometer (APD2000 pro, GNR, Italy, CRYSTAL IMPACT software 4, Bonn, Germany) with Cu-Kα1 radiation, 35 kV monochromatic voltage, and a 25 mA electric current. The range of the 2 θ diffraction angle was 4.95° to 79.75°.

The FTIR was carried out to determine the interactions of the components of the formulation, focusing on the stability of the suggested system. The Hes-NPs, drug-free nanoparticles, and Hes powder were been examined using a (BRUKER, Billerica, MA, USA) FTIR spectrometer.

### 4.4. In Vivo Experiments

#### 4.4.1. Animals

A total of 70 male albino mice (22–25 g) were obtained from the national research center in Cairo’s animal house. The mice were kept at an animal shelter at Tanta University’s Faculty of Pharmacy, where conditions included a 12 h light/dark cycle and a temperature of 25 °C. The animals had unrestricted access to food and water during the experiment. Before the trial, the animals were acclimated for seven days. The study was approved by the Research Ethics Committee of Tanta University’s Faculty of Pharmacy and followed the guidelines set out by the International Organizations Council for Medical Sciences (CIOMS) (Code of Protocol: TP/RE/5/23 p-0067).

#### 4.4.2. Experimental Design

Seven groups of animals, each containing six mice, were used: (1) Vehicle control-group mice were given a DMSO (50%)/PEG (30%)/Saline (20%) vehicle orally for 14 days. (2) Polymer control-group mice were given chitosan polymer orally for 14 days. (3) Hesperidin-group (Hes) mice were given Hes (100 mg/kg body weight) dissolved in 0.5 mL of vehicle orally for 14 days [120]. (4) Nano-hesperidin-group (Hes-NPs) mice were given Hes-NPs (100 mg/kg body weight) dissolved in 0.5 mL of vehicle orally for 14 days. (5) Doxorubicin (DOX)-group mice were given a single dose of DOX (15 mg/kg) injected intraperitoneally (IP) on the 12th day [121], with some modifications. (6) Hes-DOX-group mice were given Hes (100 mg/kg body weight) dissolved in 0.5 mL of vehicle orally for 14 days and a single dose of DOX (15 mg/kg, IP) on the 12th day. (7) Hes-NPs-DOX-group mice were given Hes-NPs (100 mg/kg body weight) dissolved in 0.5 mL of vehicle orally for 14 days and a single dose of DOX (15 mg/kg, IP) on the 12th day.

#### 4.4.3. Blood Samples and Kidney Tissue Collection

After administering DOX for 48 h [122], the mice were anesthetized with isoflurane, and blood samples were taken. Afterwards, mice were euthanized via cervical dislocation. To estimate renal functions, blood samples taken from the mice were centrifuged at 3000 rpm for 10 min using a Sigma 2-16KL centrifuge to separate the serum. The kidneys of the mice were removed. A portion of the tissue was fixed using a 10% buffered formalin solution for use in immunohistochemical and histopathological analyses. The tissue samples that were left over for the biochemical analysis were stored at −80 °C.

#### 4.4.4. Kidney Function Estimation

Serum samples were analyzed for urea and creatinine serum using kinetic methods following the manufacturer’s instructions for kits provided by SPINREACT, Santa Coloma Bas, Spain, Cat No. BSIS33-P and BSIS13-E, respectively.

#### 4.4.5. Lipid Peroxidation and Antioxidant Enzyme Activity (CAT and SOD) Estimated in Kidney Tissue

Lipid peroxidation (MDA), catalase activity (CAT), and superoxide dismutase (SOD) activity in the homogenate collected from mice kidney tissue were evaluated using commercial ELISA kits (phosphate buffer saline, pH = 7.2) provided by MyBioSource Co., San Diego, CA, USA, and CUSABIO Co., Houston, TX, USA Cat No.: MBS268427, CSB-E08556m, and CSB-E08555, respectively. Every experimental protocol followed the guidelines provided by the manufacturer.

#### 4.4.6. Determination of TNF-α, IL-1β, and VEGF Content

To estimate the content of inflammatory biomarkers (TNF-α and IL-1β) in the homogenate (phosphate buffer saline, pH = 7.2) collected from mice kidney tissue, commercial ELISA kits provided by MyBioSource Co., San Diego, CA, USA, and CUSABIO Co. Houston, TX, USA were used, with Cat No. CSB-E04741m and CSB-E08054m for TNF-α and IL-1β, respectively, following the guidelines provided by the manufacturers. In addition, the VEGF content was measured in kidney tissue homogenate using VEGF ELISA Kit PicoKine®, Boster Biological Technology, Pleasanton, CA, USA, Cat No. EK0540, following the manufacturer’s instructions.

#### 4.4.7. Quantitative Estimation of Gene Expression of *Sirt-1*, *Bcl-2*, *VEGF*, *HIF1-α,* and *Kim-1* Using Real-Time PCR (qRT-PCR)

Using *B-actin* as a housekeeping gene in qRT-PCR, the relative gene expressions of *Sirt-1*, *Bcl-2*, *VEGF*, *HIF-1-α*, and *Kim-1* were assessed. Table 3 includes a list of primer sequences. Total RNA was extracted using the TRIzol reagent (15596026) (Life Technologies, Thermo Fisher Scientific, Lenexa, KS, USA).

QuantiTect’s Reverse Transcription Kit (Qiagen, Hilden, Germany) was used to execute the reverse transcription procedure. Complementary DNA amplicons, primers, and SYBR Green Master Mix (Maxima SYBR Green/qPCR Master Mix, Thermo Fisher Scientific, Lenexa, KS, USA) were included in the reaction mixes. The gene expression relative to the calibrator control group was calculated using the fold-change method (2^−ΔΔCt^) [123].

**Table 3:** Primer sequences.

Gene	Primer Sequence (5′–3′)	Reference
***Sirt-1***	**CAC-CAG-AAA-GAA-CTT-CAC-CAC-CAG** **ACC-ATC-AAG-CCG-CCT-ACT-AAT-CTG**	[124]
***Bcl-2***	**CACCCCTGGCATCTTCTCCTT** **AGCGTCTTCAGAGACAGCCAG**	[125]
***VEGF***	**GGCTCTGAAACCATGAACTTTCT** **GCAGTAGCTGCGCTGGTAGAC**	[126]
***HIF1-α***	**GGACGATGAACATCAAGTCAGCA** **GGAATGGGTTCACAAATCAGCAC**	[79]
***Kim-1***	**CGGTGCCTGTGAGTAAATAGAT** **CTGGCCATGACACAAATAAGAC**	[80]
***B-actin***	**GTG GGA ATT CGT CAG AAG GAC TCC TAT GTG** **GAA GTC TAG AGC AAC ATA GCA CAG CTT CTC**	[81]

#### 4.4.8. Histopathological Examination

Sections of kidney tissue (3–5 µm thick) were arranged and stained with hematoxylin and eosin (H&E). Their characteristic histopathological features were examined under light microscopy. The grading system for evaluating tubular necrosis, loss of brush border, cast formation, and tubular dilatation was applied to 10 randomly selected, non-overlapping fields at 200× magnification.

#### 4.4.9. Immunohistochemical Examination

The immunohistochemical-staining method was applied according to the method described in [82]. To retrieve antigens, dewaxed sections were placed in a 0.05 M, pH 6.8 citric acid buffer solution. Afterwards, the sections were treated with protein blocks and 0.3% H_2_O_2_. Following that, the samples were incubated with the following antibodies: BAX (Santa Cruz, CA, USA, Cat No. sc-7480, 1:100 dilution), Bcl-2 (Abcam, Cambridge, UK, Cat No. ab182858, USA, 1:100 dilution), Caspase-3 (Invitrogen, Thermo Fisher Scientific, Lenexa, KS, USA, Cat No. PA5-77887, dilution 1/100), p53 (Santa Cruz, CA, USA, Cat No. sc-126, 1:100 dilution), NF-κB (Santa Cruz, CA, USA, Cat No. sc-166416, 1:100 dilution), and TGF-1β (Santa Cruz, CA, USA, Cat No. sc-130348, 1:100 dilution). Then, the secondary antibody conjugated to horseradish peroxidase was used for half an hour at 37 °C. The slides were treated three times with phosphate buffer saline after each procedure. The sections were subjected to the 3,3′-diaminobenzidine tetrahydrochloride reagent for three minutes. Ultimately, the slides underwent a counterstaining process using Mayer’s hematoxylin, followed by a distilled water wash and DPX mounting. Digital micrographs were obtained, and slides were examined under a microscope, using an Olympus CX21 (Tokyo, Japan) digital camera mounted to the microscope. The staining intensity of the kidney tissues was evaluated using a scale from 0 to 3, where 0 indicated no staining, 1 represented weak staining, 2 denoted moderate staining, and 3 signified strong staining. The extent of the stained area was assessed on a 4-point scale: 0 for 0%, 1 for 25%, 2 for 25–60%, and 3 for more than 60%. The total immunoreactivity score (IRS) for each tissue section was calculated by summing the intensity and area scores [82].

#### 4.4.10. Statistical Analysis

Data are presented as the mean values ± standard deviation (SD). Tukey’s multiple comparisons were used after one-way ANOVA to ascertain the differences between the groups. A *p*-value of less than 0.05 was employed to demonstrate statistical significance. A GraphPad Prism, version 5 (GraphPad Software Inc., La Jolla, CA, USA), was used to perform statistical computations.

## 5. Conclusions

This study revealed that Hes-NP treatment diminishes renal toxicity induced by DOX by alleviating oxidative stress and inflammation and reducing apoptosis. Such observations are important as they indicate that Hes-NPs could be used as a therapeutic approach for managing renal toxicity prompted by DOX. Furthermore, their anti-apoptotic potential could be beneficial for the treatment of cancer, which requires further research. Nevertheless, future investigations should be performed to reveal clinical data on the role of Hes-NPs in protecting against renal injury. This would provide a scientific basis for the use of Hes-NPs as a powerful antioxidant, anti-inflammatory, and anti-apoptotic agent for the attenuation of DOX-triggered renal toxicity.

## Figures and Tables

**Table 1:** Polymeric Hes-NP characteristics.

Parameter	Mean	Range
% Entrapment efficiency	83.00 ± 7.3	77.27–91.34
% Yield	31.16 ± 1.4	25.0–29.8
Particle size (nm)	127 ± 32.15	107.4–164.1
Zeta potential (mV)	−51.12 ± 9.79	−60.26–−38.37

**Table 2:** Immunohistochemical marker scores across experimental groups *.

Group	Average of Total Immunoreactivity Score (IRS)				
	Caspase-3	NF-κB	TGF-β	BAX	Bcl-2
Vehicle Control	0.0	0.0	0.4	0.4	5.2
Polymer Control	0.4	0.0	0.4	0.4	4.8
Hes	0.4	0.4	0.0	0.4	5.0
Hes-NPs	0.4	0.0	0.0	0.4	5.0
DOX	4.6	4.4	3.8	5.0	2.5
Hes-DOX	3.6	2.6	2.0	3.2	3.4
Hes-NPs-DOX	2.4	1.6	1.2	2.0	4.4

* Immunohistochemical scoring: Staining intensity: 0 (no staining), 1 (weak), 2 (moderate), 3 (strong). The extent of stained area: 0 (0%), 1 (≤25%), 2 (26–60%), 3 (>60%). Total score (IRS): Sum of intensity and extent scores.

## Data Availability

Data are contained within the article.
